# Severe hydrops in the infant of a Rhesus D-positive mother due to anti-c antibodies diagnosed antenatally: a case report

**DOI:** 10.1186/1752-1947-4-57

**Published:** 2010-02-18

**Authors:** Shilpa Singla, Sunesh Kumar, Kallol Kumar Roy, Jai Bhagwan Sharma, Garima Kachhawa

**Affiliations:** 1 Department of Obstetrics and Gynecology, All India Institute of Medical Sciences, New Delhi, 110029, India

## Abstract

**Introduction:**

Rhesus haemolytic disease of the newborn is a prototype of maternal isoimmunisation and fetal haemolytic disease. There are other rare blood group antigens capable of causing alloimmunisation and haemolytic disease such as c, C, E, Kell and Duffy. In India, after the confirmation of a newborn's blood group, antibodies are screened only if the mother is Rehsus D-negative negative and the father is Rhesus D-positive. Hydrops in Rhesus positive women are investigated along the lines of non-immune hydrops.

**Case presentation:**

We report the case of a patient from India where irregular antibodies were requested for an O-positive 26-year-old mother in order to investigate fetal hydrops. Anti-c antibody was revealed and the fetus was treated successfully with compatible O negative and c negative intrauterine blood transfusions. The baby was treated postnatally with double volume exchange transfusion with the same compatible blood, and was discharged 30 days after birth.

**Conclusion:**

We highlight the importance of conducting irregular antibody screening for women with significant obstetric history and fetal hydrops. This could assist in diagnosing and successfully treating the fetus with appropriate antigen negative cross-matched compatible blood. We note, however, that anti-c immunoglobulin is not yet readily available.

## Introduction

Haemolytic disease of the newborn is a well-recognised entity because of the isoimmunisation of Rhesus D-negative mother in an Rh-positive fetus. Severe degrees of fetal hemolysis result in fetal hydrops [1]. Although anti-Rh(D) was once the major etiology of haemolytic disease of the fetus and newborn (HDFN), the widespread adoption of antenatal and postnatal Rhesus immunoglobulin has resulted in a marked decrease in the prevalence of alloimmunisation due to the RhD antigen present during pregnancy. Maternal alloimmunisation to other red cell antigens remains the cause of fetal disease since no prophylactic immunoglobulins are available to prevent the formation of these antibodies [2].

Mild to severe cases of fetal haemolytic disease have been reported when anti-c, C, e, E, or Kell, Kidd, Duffy, MNS, Lutheran, Diego, Xg, P antibodies, as well as other private and public blood group systems found in the sera of mothers [3]. It is recommended that routine red cell antibody screening be done at the first appointment in pregnant mothers and, if no antibodies are detected, once more in the third trimester between 28 and 36 weeks [4]. The guidelines state that further testing is unnecessary, since immunisation during late pregnancy is unlikely to result in an antibody concentration that would be sufficient to cause severe haemolytic disease of the neonate [4].

However, in the majority of transfusion and antenatal care centres in India and other developing countries, routine antenatal antibody screening is done only for Rh(D)-negative mothers to screen for Anti-D antibodies. Hence, there may be a serious delay in diagnosing HDFN due to the rarity of antigens [5]. The first case of haemolytic disease of the newborn due to anti-c antibodies in India was published in a retrospective diagnosis made in 2007. Fetal affection was noted to be of a milder variety, where the baby was managed only with intravenous immunoglobulin and phototherapy [5].

Here we report the first known case of antenatally diagnosed anti-c antibodies that resulted in severe fetal hydrops, thus requiring multiple *in utero *and *ex utero *transfusions.

## Case presentation

A 26-year-old Indian woman was admitted to our gastroenterology unit with extrahepatic portal vein obstruction with features of massive malena at 29 weeks of gestation. She had a previous pregnancy that resulted in a single offspring. She was referred for an antenatal check-up to our obstetric unit, where after clinical examination, an ultrasonography was performed which revealed gross fetal hydrops. She was transferred to our obstetric unit for further evaluation and management.

Her prenatal course was complicated by recurrent episodes of hematemesis and malena for 10 years prior to admission and she was previously diagnosed with esophageal varices. She had a history of multiple blood transfusions and sclerotherapy sessions. Her first pregnancy was 2 years prior to admission, in which she had regular supervised antenatal checkups on her first and second trimesters with a normal anomaly scan. Her pregnancy was complicated by gestational diabetes mellitus that was controlled through diet. She had episodes of recurrent malena in this pregnancy. Her third trimester was unsupervised at home and she was admitted to a local private practitioner at the onset of her labour. She underwent caesarean section for meconium-stained liquor. She delivered a grossly normal male baby with jaundice at birth. Details pertaining to the baby are not available, but the baby had received a blood transfusion on the third day of life and died on the seventh day.

Her present pregnancy was a spontaneous conception. Her first and second trimester antenatal checkups were with a private practitioner in her hometown. She presented in the gastroenterology department of our institute at 29 weeks of gestation with massive malena and anemia. Upper gastrointestinal (GI) endoscopy revealed the presence of grade II esophageal varices for which sclerotherapy was done. She was given three units of packed red blood cells to raise her haemoglobin from 6 gm% to 10 gm%. Upon obstetric referral, ultrasonography at 30 weeks and 5 days revealed severe fetal hydrops. Doppler studies were suggestive of fetal anemia. She was given corticosteroids for fetal lung maturity. At 31 weeks, cordocentesis was done and intravascular intrauterine fetal transfusion with O-negative cross-matched blood was given. Since she was Rhesus (D) O type positive, non-immune hydrops was suspected. Fetal blood was thus sent for blood grouping, haemoglobin, haematocrit, TORCH serology, parvovirus B19 serology, haemoglobin electrophoresis, G6PD enzyme assay and karyotype.

The results of fetal echocardiography were normal. Fetal blood group was A-positive and was negative in workup for non-immune hydrops. Maternal serum indirect Coomb's test was positive, thus leading to a suspicion of the presence of non-anti-D antibodies. A special request was sent to the blood bank to screen for uncommon rare blood groups and non-D antibodies. Anti-c was detected in maternal serum in 1:4 dilutions.

Maternal blood was resent to the blood bank to establish exact Rhesus haplotype, which was determined to be R1R1 (CDe/CDe). The fetus was monitored with bi-weekly biophysical profiling and a second intrauterine transfusion was given one week later with compatible O-negative and c-negative cross-matched blood. At 32 weeks and 5 days, an emergency caesarean section was done due to poor biophysical profile.

A grossly hydropic female baby with a birth weight of 1.6 kg was born with massive ascites, hepatosplenomegaly, pallor and hypotonia. At birth, 150 ml of ascitic tap was done and the baby was transferred to the nursery on bag and tube ventilation. Her cord blood hematocrit was 15 and total serum bilirubin was 6.5 mg/dl. A partial exchange transfusion followed by double volume exchange transfusion with O-positive, c-negative blood was performed. Intravenous immunoglobulin in doses of 1 gram/kg was administered after the exchange. The baby required intense phototherapy for 4 days. By the second week, she developed conjugated hyperbilirubinemia due to inspissated bile syndrome, which resolved on its own. The baby was then managed conservatively and was discharged in stable condition on the 30th day of her life.

The fetal rhesus haplotype was established finally as cDe/cDe. The source of maternal isoimmunisation may be either fetomaternal haemorrhage in the mother s current or previous pregnancy or from multiple blood transfusions.

## Discussion

Fetal hydrops may be classified as either immune or non-immune. Non-immune hydrops result from reasons other than antigen to antibody incompatibility. Immune hydrops results from maternal antibodies that are capable of crossing the placenta to react with fetal antigen, thus causing a reaction that manifests as fetal haemolysis. In the largest retrospective review on the etiology of hydrops [6] involving 598 patients, the cause of hydrops was established unclearly in 26.3% of cases, isoimmunisation in 4.5% of cases, and non-immune etiology in 69.2% of cases. When isoimmunisation is identified as the cause, Rhesus antigens were responsible for 4.2% of cases. There were only two cases due to non-Rh antigens, one each due to Kell and to Duffy [6].

Out of 49 known Rhesus antigens, only 5 (c, C, D, e, E) are well known. There is no d antigen. Besides this, there are other rare blood group systems like Kell, Duffy (Fya and FYb), Kidd (JKa and JKb), and the M and N system [7].

CDe is the most common haplotype in Caucasians (42%), Native Americans (44%) and Asians (70%) [8]. In various retrospective studies, haemolytic disease of the newborn has been reported due to Anti-c antibodies [9-11]. The frequency of D and Non-D antigens differ in different populations with respect to their ethnic origin [12-14].

It is important to check that patients with anti-c antibodies do not have any additional antibodies for although these might not compound any HDFN, they would certainly delay the provision of compatible blood for transfusion [9]. Bowell *et al*. (1986) observed a strong co-association of anti-c with anti-E in 41% of their subject population [9]. Perinatal mortality due to anti-c alone is usually much lower than that of Anti-D (1 in 250,000 versus 1 in 25,600 births, respectively) [15].

However, the combination of anti-c and anti-E antigens can cause the occurrence of severe fetal and neonatal haemolytic disease [3].

The management of anti-c isoimmunisation or isoimmunisation with any other irregular red blood cell antibody is similar to the management of anti-D isoimmunised pregnancy, with a specification that blood used for fetal and/or neonatal transfusion should be negative for its respective antibody. Critical titre needs to be standardized with respect to individual laboratories. In a series by Hackney *et al*. [11], a critical titre of 1:32 without supplementation with ultrasound and a critical titre of 1:16 supplemented with ultrasonographic features of hydrops were considered significant. In our patient, a dilution titre of 1:4 was associated with fetal hydrops and no other irregular antibody or any other cause of non-immune hydrops could be attributed. There was a significant improvement in fetal and neonatal anemia following transfusion with c-negative blood, attributing it as the sole cause of fetal haemolysis and hydrops. However, fetal and/or neonatal direct Coomb s test (DCT) positivity does not necessarily correlate with a severe haemolytic disease. DCT may be negative in anti-c isoimmunisation as this antibody is often present in small titres [9]. Serologic evaluation of maternal antibodies thus remains the cornerstone of management as approximately only 50% of antigen positive fetuses require a more invasive testing [11].

Our experience in the management of our patient was similar to that of other authors [3,5,11]. However, instead of the traditional method of spectrophotometric evaluation of amniotic fluid OD 450 to assess fetal bilirubin due to severity of haemolysis, anemia was assessed using the more modern method of colour Doppler ultrasound and of plotting peak systolic velocity of the middle cerebral artery on Mari charts [16]. However, fetal anemia may not have been severe enough to be detected by Mari s criteria of the middle cerebral artery peak velocity, which highlights the usefulness of the traditional Liley curve and the Queenan charts.

Through this case report, we want to emphasize the potential developing immune hydrops due to irregular red blood cell antibodies. This contribution to the literature is of particular importance since there is an observed paucity of data on the prevalence of irregular antibodies in the Indian population, despite having one of the highest obstetric loads in the world.

## Conclusions

There is a need to impose properly formulated protocols to screen pregnant women with unfavourable obstetric history of late trimester mishaps and pregnancies with fetal hydrops. Blood bank guidelines for the screening of maternal serum antibodies and facilities have to be updated to decrease the occurrence of preventable perinatal morbidity and mortality.

## Abbreviations

DCT: direct Coombs test; GI: gastrointestinal; G6PD: Glucose 6 phosphate dehydrogenase; HDFN: haemolytic disease of the fetus and/or newborn; OD: optical density.

## Consent

Written informed consent was obtained from the patient and from the mother on behalf of the infant for publication of this case report and any accompanying images. A copy of the written consent is available for review by the Editor-in-Chief of this journal.

## Competing interests

The authors declare that they have no competing interests.

## Authors' contributions

SS assisted in the antenatal care of the mother, carried out the literature search and wrote the manuscript. SK, KR, JBS and GK helped in managing the case and in providing the final draft of the manuscript. All authors read and approved the final manuscript.
